# Sintering and Tribological Properties of Ti_3_SiC_2_-TiSi_x_ Composite Sintered by High-Pressure High-Temperature Technology

**DOI:** 10.3390/ma17194866

**Published:** 2024-10-03

**Authors:** Yuqi Chen, Liang Li, Ming Han, Chaofan Sun, Jin Li

**Affiliations:** 1 Intelligent Manufacturing and Electrical Engineering, Nanyang Normal University, Nanyang 473061, China; 2 Henan Academy of Science, Zhengzhou 450002, China

**Keywords:** Ti_3_SiC_2_-TiSi_x_ composite, high-temperature technology, tribological properties

## Abstract

The Ti_3_SiC_2_TiSi_x_ ceramic composite was synthesized in situ from a mixture of 3Ti:1.5Si:1.2C powders under pressures ranging from 2 to 5 GPa and temperatures of 1150 °C to 1400 °C. At medium and high temperatures (4–5 GPa and 1400 °C), Ti_3_SiC_2_ dissolves into the cubic TiC phase. SEM analysis revealed that the high-pressure-produced multilayer structure of Ti_3_SiC_2_ remained intact. The friction properties of Ti_3_SiC_2_-TiSi_x_ composites combined with copper and aluminum were studied under both dry and lubricated conditions. After the break-in period, the Ti_3_SiC_2_-TiSi_x_/Al combination exhibited the lowest friction coefficient: approximately 0.2. In dry-sliding conditions, the friction coefficient varies between 0.5 and 0.8. The wear mechanisms for Ti_3_SiC_2_-TiSi_x_ composites paired with aluminum primarily involve pear groove wear and adhesive wear during dry friction. Irregularly shaped aluminum balls accumulate in the pear grooves and adhere to each other. With increasing sintering pressure, the average friction coefficient of Ti_3_SiC_2_-TiSi_x_ composites against Cu ball pairs first increases and then decreases. The wear rate of the samples did not vary significantly as the sintering pressure increased, whereas the wear rate of Cu balls decreased with increasing sintering pressure. The adhesive wear of the Ti_3_SiC_2_-TiSi_x_ composite with its Cu counterpart is stronger than that of the Al counterpart. Abrasive chips of Cu balls appeared in flake form and adhered to the contact interface.

## 1. Introduction

The ternary lamellar ceramic Ti_3_SiC_2_ offers a remarkable combination of the superior properties found in both ceramics and metals [1]. As a result, it has wide-ranging applications, including use as a binder for super-hard materials [2,3,4,5], in copper matrix composites [6,7,8], and in ceramic composites [9,10,11,12,13,14,15]. The unique properties of Ti_3_SiC_2_ have spurred significant research into its preparation and application, with a focus on understanding the fundamental principles of the microstructure–property relationship [16,17]. The properties are highly dependent on the microstructures, which are influenced by the synthesis method. The most commonly used method is hot pressing, applying approximately 35 MPa to produce dense Ti_3_SiC_2_ solids [9].

Spark plasma sintering (SPS) at pressures ranging from 50 MPa to 80 MPa [11] and hot isostatic pressing (HIP) at approximately 200 MPa have been shown to enhance the microstructure and performance of Ti_3_SiC_2_. High sintering pressure, on the order of several GPa, facilitates the synthesis of dense Ti_3_SiC_2_, particularly in super-hard composites. Some studies have successfully synthesized diamond or cBN composites with Ti_3_SiC_2_ and related MAX-phase compounds at pressures exceeding 3 GPa [2,3,4,5,18]. The ternary Ti–Si–C alloy phase diagram has been investigated for the synthesis of Ti_3_SiC_2_ at temperatures ranging from 1250 to 2877 °C. However, there are conflicting conclusions regarding the synthesis and stable phase region of Ti_3_SiC_2_ under high pressure. Qin et al. [19] reported that Ti_3_SiC_2_ powders become unstable between 3 and 5 GPa, with Ti_3_SiC_2_ breaking down into TiC at temperatures above 1000 °C under 3 GPa. The critical disintegration pressure and temperature change linearly. Meng et al. [20] investigated the formation mechanism of Ti_3_SiC_2_ with different raw material types (Si/SiC or TiC/C) and proportions. Excess silicon is beneficial for the synthesis of high-purity Ti_3_SiC_2_ at 1300 °C. Depending on the ratio of starting materials, reactions in the Ti-Si-C system may yield TiC, SiC, TiS_2_, Ti_5_Si_3_C, or Ti_3_SiC_2_ [21]. The influence of sintering pressure on the formation mechanism is essential for further study. TiSi_2_, as an intermediate phase in the synthesis of Ti_3_SiC_2_, indirectly reflects how different temperatures and pressures affect the stability of Ti_3_SiC_2_.

To prevent the decomposition of Ti_3_SiC_2_ caused by the diffusion of silicon atomic layers under high pressure, Zhu et al. [3] employed a high-pressure, high-temperature sintering process to synthesize polycrystalline diamond, using Ti_3_SiC_2_ and Si as the binder. The Ti_3_SiC_2_-Si binder composite began to decompose into TiC and SiC at temperatures above 1350 °C under 5.5 GPa. Among the Ti_3_SiC_2_-based composite materials, most studies have focused on Ti_3_SiC_2_-TiC [22] or Ti_3_SiC_2_-SiC [9,10,12] composites, with only limited research on Ti_3_SiC_2_-TiSi_x_ composites. TiSi_2_ is an excellent reinforcement material due to its high melting point (1540 °C), oxidation resistance, and mechanical stability [23]. Additionally, it is widely used in the semiconductor industry for its outstanding electrical properties [24,25].

The study of TiSi_2_ matrix composites prepared under high pressure provides indirect insights into the stability of Ti_3_SiC_2_. The mechanism of Ti_3_SiC_2_ synthesis under high pressure, in the presence of abundant titanium and silicon, was investigated. The effect of raw material composition (3Ti:1.5Si:1.2C) was explored in comparison to previous studies. Additionally, the friction properties of Ti_3_SiC_2_-TiSi_x_ composites synthesized under varying pressures were examined.

## 2. Materials and Methods

To synthesize Ti_3_SiC_2_-TiSi_x_ composites, titanium (Ti, 325 mesh, 99.3 wt.% purity), silicon (Si, 325 mesh, >99.7 wt.% purity), and graphite (325 mesh, 99% purity) powders were measured in a molar ratio of 3:1.5:1.2. The powders were then mixed in a mixer at 300 rpm/min for 12 hrs. The mixture was compacted into tablets with a diameter of 14 mm and a height of 5 mm using a cemented carbide die.

**Figure 1:**
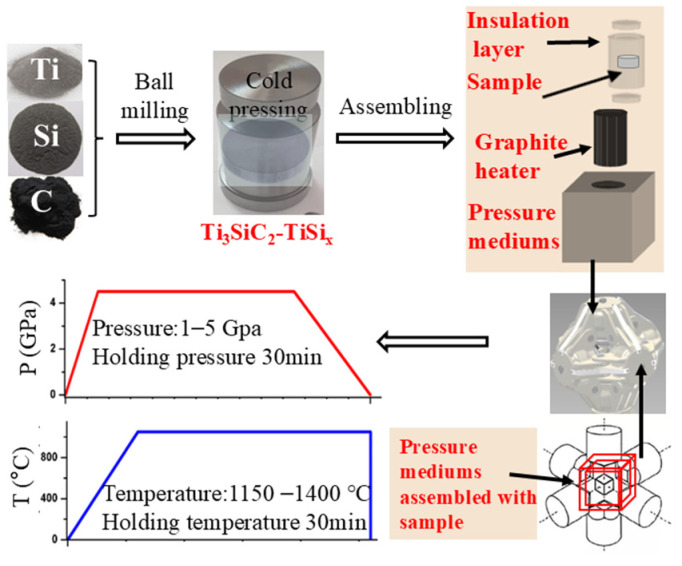
The experimental procedure of high-pressure high-temperature sintering technology.

Ti_3_SiC_2_-TiSi_x_ samples were prepared using a high-pressure sintering (HPS) apparatus (SPD 6× 1200, Xianyang Superhard Materials Equipment (Group) Co., Ltd., Xian, China) at temperatures ranging from 1150 °C to 1400 °C and pressures between 1 and 5 GPa. The temperature and pressure were increased over a period of approximately 3 min. Both were maintained for a predetermined holding time. Upon completion of the holding time, the power was immediately cut off, and the pressure was gradually released over a period of about 15 min. The sintering process followed the procedure described in previous studies [4,5]. A schematic representation of the high-pressure and high-temperature sintering process is provided in Figure 1.

The composition of the sintered compacts was analyzed using X-ray diffraction (XRD, Brukeraxs Co., Karlsruhe, Germany). The X-ray utilized a copper (Cu) target, with a loading voltage of 40 kV and a current of 40 mA. The cross-section morphology and microstructure of sintered Ti_3_SiC_2_-TiSi_x_ composites was examined using scanning electron microscopy (SEM, JSM-6390LV, JEOL, Tokyo, Japan).

The friction and wear tests of sintered Ti_3_SiC_2_-TiSi_x_ bulks were conducted using the pin-on-disk-type CFT-I material surface performance tester (Zhongke Kaihua Instrument Equipment Co., Ltd., Beijing, China). The surface of the Ti_3_SiC_2_-TiSi_x_ sample was treated with sandpaper prior to the test, achieving a surface roughness of approximately 0.1 mm. Small differences in surface roughness or variations in material composition can lead to discrepancies in friction and wear results. Typically, the surface roughness and compositional homogeneity of samples synthesized at high temperatures and pressures are consistent. The Ti_3_SiC_2_-TiSi_x_ sample was secured using hollow disk bolts under compression. The counter-abrasives were aluminum or copper balls with a diameter of Φ 4 mm. The test balls and samples were cleaned ultrasonically with alcohol before testing. The dry friction and wet grinding reciprocating sliding tests were conducted at room temperature with a sliding distance of 5 mm, a drive motor speed of 300 rpm/min, a load of 12 N, and a test duration of 30 min. The dynamic real-time friction coefficient was automatically recorded by the computer during the test. Fluctuations in the load force were due to vibrations in the drive mechanism. The variation in the error between the actual value and the set value of the load with time is illustrated in Supplementary Figure S1.

## 3. Results

### 3.1. XRD Results of Ti_3_SiC_2_-TiSi_x_ Composite

**Figure 2:**
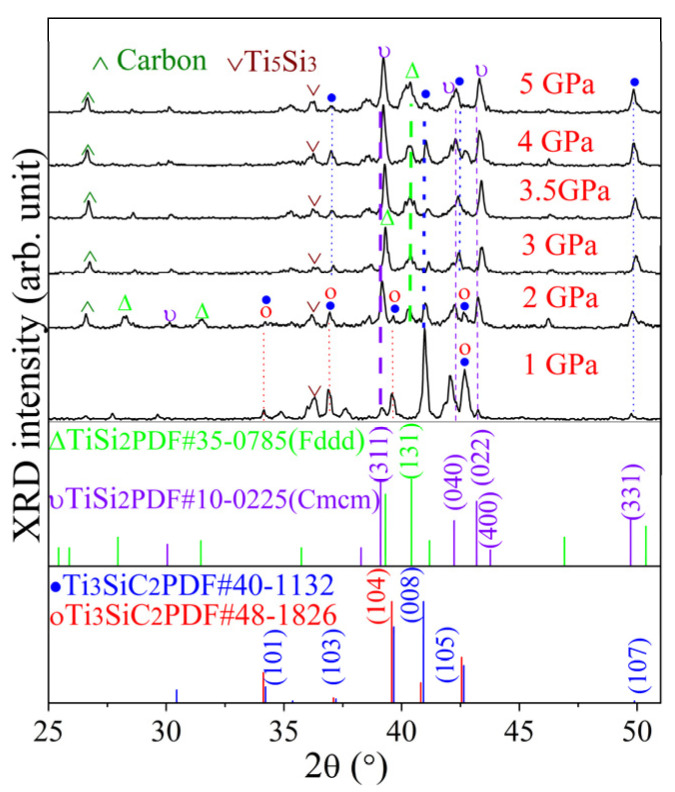
XRD patterns of Ti_3_SiC_2_-TiSi_x_ composites sintered under 1~5 GPa at 1150 °C for 30 min.

Figure 2 presents the XRD patterns of samples synthesized from mixtures containing a molar ratio of 3Ti/1.5Si/1.2C under pressures ranging from 1 to 5 GPa for 30 min at 1150 °C. At 1 GPa, distinct Ti_3_SiC_2_ peaks are visible, as shown in Figure 2. With an increase in synthetic pressure to 2–3 GPa, the prominent peaks of Ti_3_SiC_2_ and the intermediate phase Ti_5_Si_3_ diminished. Concurrently, the TiSi_2_ phase and residual carbon formed. At 3.5 GPa, the intensity of the distinctive Ti_5_Si_3_ distinctive peaks was at its lowest, and nearly vanished. Interestingly, Ti_5_Si_3_ peaks increased when the synthesizing pressure rose to 4 GPa, then decreased again as the pressure increased to 5 GPa. Between 2 GPa and 5 GPa, TiSi_2_ became the predominant phase, with its characteristic peaks shifting towards smaller angles. TiSi_2_ crystallizes in an orthorhombic structure with Cmcm (C49) and Fddd (C54) [26]. The TiSi_2_ phase was stable, exhibiting a contraction of the lattice constant of about 0.01 during pressurization from 0 to 5 GPa [27]. There is the possibility of the formation of solid solutions of orthorhombic TiSi_2_; however, hexagonal TiSi_2_ was not observed. This result is analogous to the reaction where TiC starts to react with Si, forming the TiSi_2_ phase at 1150 °C under 2 GPa [28].

**Figure 3:**
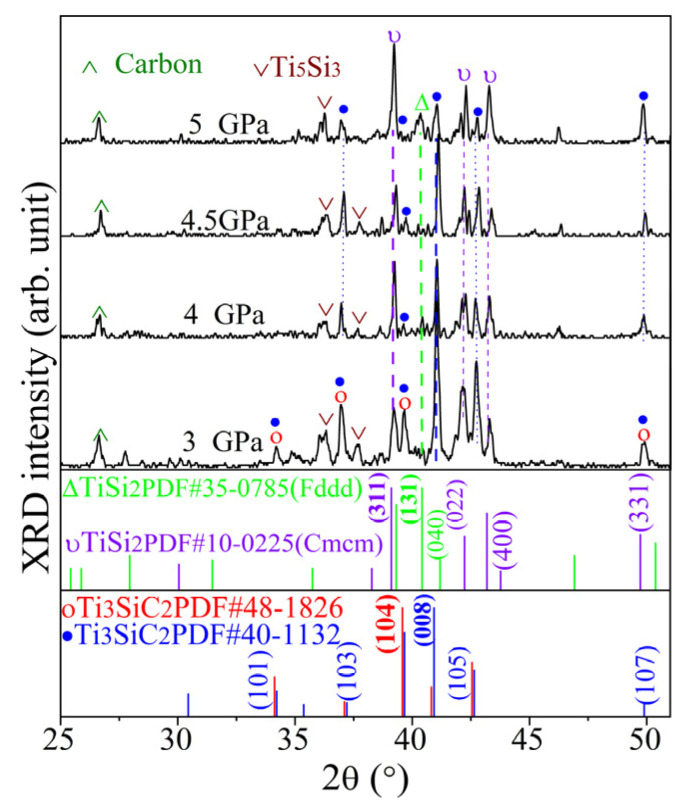
XRD patterns of Ti_3_SiC_2_-TiSi_x_ composites sintered under 3~5 GPa at 1250 °C for 30 min.

Figure 3 presents the XRD patterns of Ti_3_SiC_2_-TiSi_x_ composites synthesized from a molar ratio of 3Ti/1.5Si/1.2C at various pressures for 30 min at 1250 °C. Ti_3_SiC_2_ is the dominant phase; however, Ti_5_Si_3_ and TiSi_2_ phases coexisted under 3 GPa. This contrasts with the pulse discharge sintering conducted at a pressure of 50 MPa. The synthesis of Ti_3_SiC_2_ was achieved during sintering at temperatures of 1250 °C and higher using pulse discharge sintering [29]. At pressures exceeding 3 GPa, the sintering pressure had no impact on the chemical composition, as shown in Figure 3. However, upon increasing the pressure to 4 GPa, the characteristic peak of Ti_3_SiC_2_ (lattice plane 008) diminished. Interestingly, while the TiSi_2_ phase decreased, the Ti_3_SiC_2_ peaks increased at 4.5 GPa; the situation differed significantly at 5 GPa. The primary component synthesized at pressures ranging from 3 to 5 GPa and temperatures of 1150 °C or 1250 °C is TiSi_2_, indirectly indicating that this temperature–pressure interval is more suitable for the high-pressure preparation of TiSi_2_.

**Figure 4:**
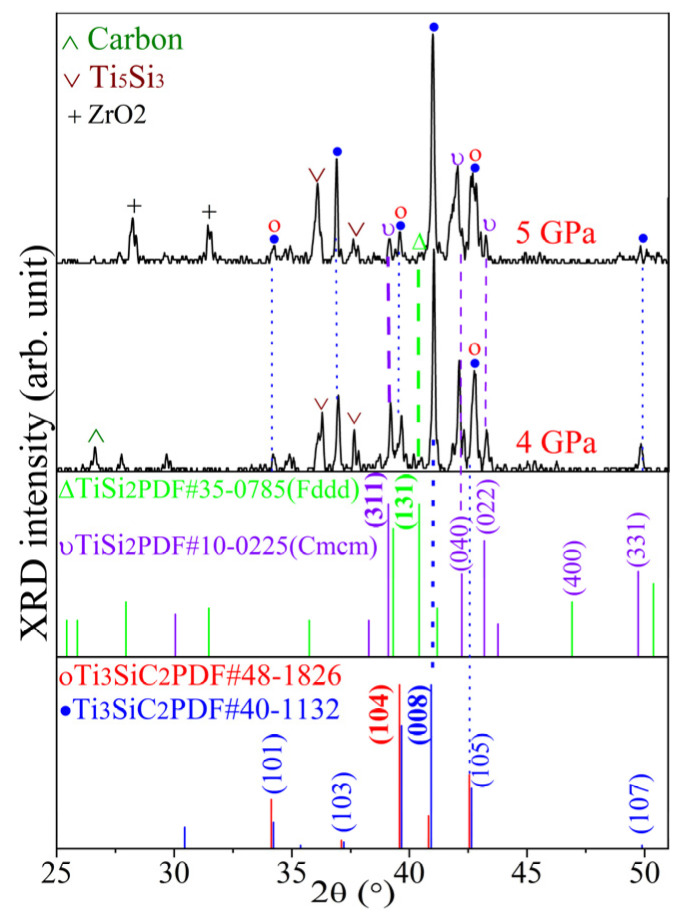
XRD patterns of Ti_3_SiC_2_-TiSi_x_ composites sintered under 4~5 GPa at 1400 °C for 30 min.

Figure 4 presents the XRD patterns of Ti_3_SiC_2_-TiSi_x_ composites fabricated from a mixture with a molar ratio of 3Ti/1.5Si/1.2C at 1400 °C under pressures of 4 to 5 GPa for 30 min. The predominant phase is Ti_3_SiC_2_, which coexists with trace amounts of TiS_2_ and Ti_5_Si_3_. The excessively high sintering pressure and temperature led to the emergence of ZrO_2_ peaks. The sintered results are comparable to those synthesized at 1150 °C under 1 GPa and 1250 °C under 3 GPa. Ti_3_SiC_2_ completely decomposes at 5 GPa and 1300 °C [19]. The enhanced stability of Ti_3_SiC_2_ can be explained in two ways: 1) the Si content of the starting material is greater than the stoichiometric ratio of Ti_3_SiC_2_; 2) the secondary-phase TiS_x_ exhibits high hardness (8.7 GPa for TiSi_2_ and 9.8 GPa for Ti_5_Si_3_) [30] and high Young’s modulus (256 GPa for TiSi_2_ and 156 GPa for Ti_5_Si_3_) [31]. The excess Si enhances the stability of the Ti_3_SiC_2_ phase under high pressure, similar to the preparation of the Ti_3_SiC_2_ phase through chemical vapor deposition (CVD) [32], arc melting [33], self-propagating high-temperature synthesis (SHS) [34], pressure-less synthesis [35], reactive melt infiltration (RMI) [36], and pulse discharge sintering (PDS) processes [37]. The excess silicon likely compensates for losses due to evaporation. Currently, there is no thermodynamic phase diagram available for the stability of the high-pressure Ti_3_SiC_2_ phase. Phase diagrams of Ti-Si-C at 1200 °C under atmospheric pressure indicate that all samples with excess silicon have comparable and relatively low amounts of TiSi_2_ [38]. When the synthesis temperature exceeds 1300 °C, silicon can form a low melting point eutectic with the TiSi_2_ alloy, as depicted in the calculated Ti-Si-C ternary phase diagram at 1400 °C and 1800 °C, respectively [39]. The hard-phase TiSi_2_ and Ti_5_Si_3_ compartmentalize the Ti_3_SiC_2_ into hermetically sealed units at high pressure, thereby inhibiting Si diffusion escape. Under high-pressure conditions, these hard phases (such as diamond or cBN) act to compartmentalize the structure, creating barriers to Si atom diffusion. Referring to the study of Ti_3_SiC_2_ as a superhard material binder, the graphical abstract in Ref. [2], Figure 4b in Ref. [40], and Figure 15 in Ref. [18] illustrate the mechanism of sealing the bonded phase with the hard phase under high pressure.

### 3.2. Microstructure of Ti_3_SiC_2_-TiSi_x_ Composites

**Figure 5:**
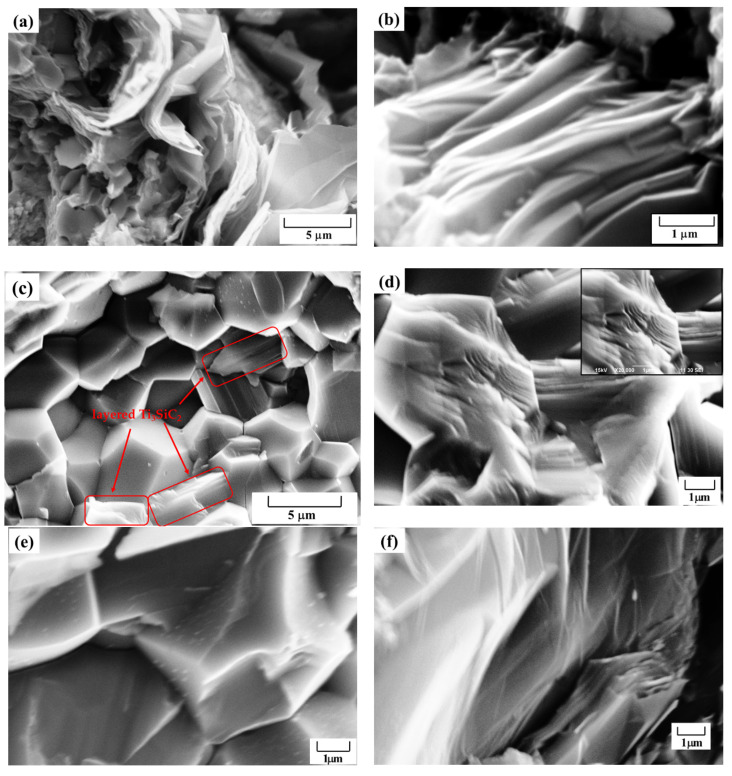
SEM images of the products from 3Ti/1.5Si/1.2C sintered at different conditions: (**a**) 1150 °C and 1 GPa; (**b**) layered structure of Ti_3_SiC_2_; (**c**) 1150 °C and 3 GPa low-magnification image; (**d**) high-magnification image of layered structure of Ti_3_SiC_2_; (**e**) high-magnification image of orthorhombic TiSi_2_; (**f**) interface between TiSi_2_ and layered Ti_3_SiC_2_ structure.

Figure 5 presents fracture surface micrographs of sintering products fabricated from 3Ti/1.5Si/1.2C under pressure at temperatures ranging from 1150 to 1400 °C for 30 min. The sample consists of Ti_3_SiC_2_, TiSi_2_, Ti_5_Si_3_, and graphite, as shown in Figure 5a. The high-pressure sintered sample in Figure 5b is nearly fully dense and exhibits the characteristic layered structure of Ti_3_SiC_2_. As deduced from Figure 2, Ti_3_SiC_2_ can be synthesized at this temperature and pressure under 1 GPa. The Ti_3_SiC_2_-TiSi_x_ composites demonstrate good interfacial bonding, akin to cBN-Ti_3_AlC_2_ composites [4] and cBN-Ti_3_SiC_2_ composites [5]. The presence of Ti_3_SiC_2_ is further confirmed by the typical layered structure observed in Figure 5b.

Figure 5c displays a low-magnification image of the sample sintered at 1150 °C under 3 GPa for 30 min. The primary phases observed include orthorhombic TiSi_2_ and layered Ti_3_SiC_2_, highlighted in the red rectangle, which aligns with the XRD result in Figure 2. TiSi_2_ adopts an orthorhombic structure, and a potential phase change under pressure during high-temperature high-pressure sintering could result in the formation of microcracks observed during the friction test. High-magnification images of the layered Ti_3_SiC_2_ structure and orthorhombic TiSi_2_ are provided in Figure 5d,e, respectively. The interaction between TiSi_2_ particles and the stacked Ti_3_SiC_2_ layers is depicted in Figure 5f.

### 3.3. Friction Behavior of Ti_3_SiC_2_-TiSi_x_ Composites

**Figure 6:**
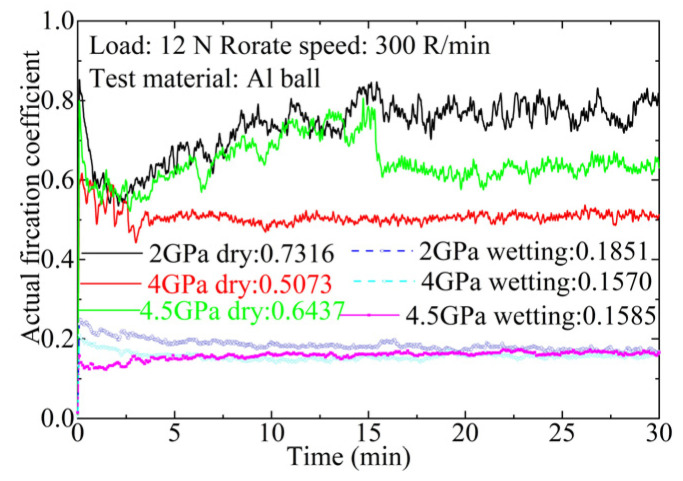
COF of Ti_3_SiC_2_-TiSi_x_ composites versus sliding time.

Figure 6 presents the friction coefficient (COF) of Ti_3_SiC_2_-TiSi_x_ composites versus sliding time. The frictional behavior is highly sensitive to the test conditions. The average COF of Ti_3_SiC_2_-TiSi_x_ composites sliding against an Al ball was measured with a normal load of 12 N.

Following the break-in period (150 s), the Ti_3_SiC_2_-TiSi_x_/Al pair exhibited the lowest friction coefficient (approximately 0.2), with minimal fluctuations during the stable period. These slight fluctuations in COF during the steady period can be attributed to the plastic deformation of stressed surfaces, and the reduction in stiffness during the wet-sliding test [41]. Notably, the COF remained unaffected by variations in sintering pressure.

The COF increases significantly under dry-sliding conditions compared to wet-sliding conditions. As the sintering pressure increases, the COF initially decreases and then rises. The COFs of Ti_3_SiC_2_-TiSi_x_ composites sintered at 2 GPa and 4.5 GPa are 0.7316 and 0.6437, respectively. These COF values for Ti_3_SiC_2_-TiSi_x_ composites are similar to those of Ti_3_SiC_2_-PbO-Ag composites tested against the Inconel 78 alloy [42]. Relatively significant fluctuations were observed during the sliding phase of both test pairings. The Ti_3_SiC_2_-TiSi_x_/Al pairing exhibited a COF of approximately 0.5073. The COF ranges from 0.5 to 0.73, depending on the chemical composition of the Ti_3_SiC_2_-TiSi_x_ composites. This suggests that, under similar testing conditions, the COF is highly sensitive to the material composition.

**Figure 7:**
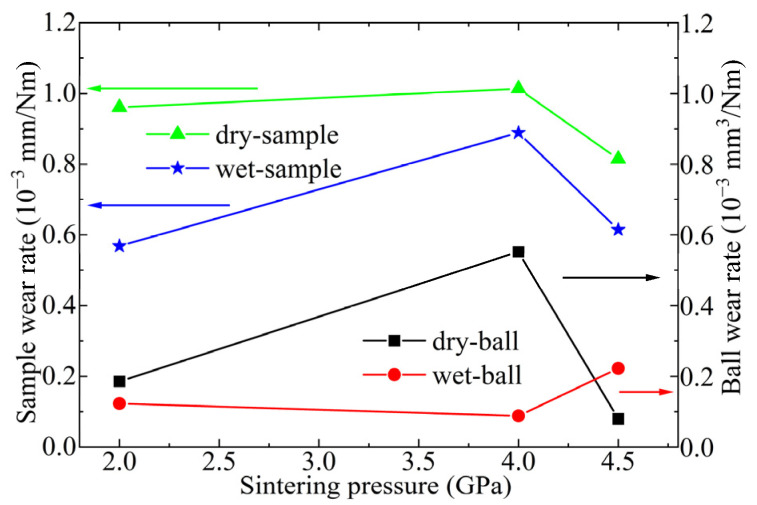
Wear rates of Ti_3_SiC_2_-TiSi_x_ composites sliding against Al ball.

The wear rates of Ti_3_SiC_2_-TiSi_x_ composites sliding against an Al ball are shown in Figure 7. Ti_3_SiC_2_-TiSi_x_ composites exhibit a higher wear rate under dry-sliding conditions. The Ti_3_SiC_2_-TiSi_x_ composites sintered at 4 GPa exhibit the highest wear rate. The wear rate of Ti_3_SiC_2_-TiSi_x_ composites initially increases and then decreases as the sintering pressure increases.

**Figure 8:**
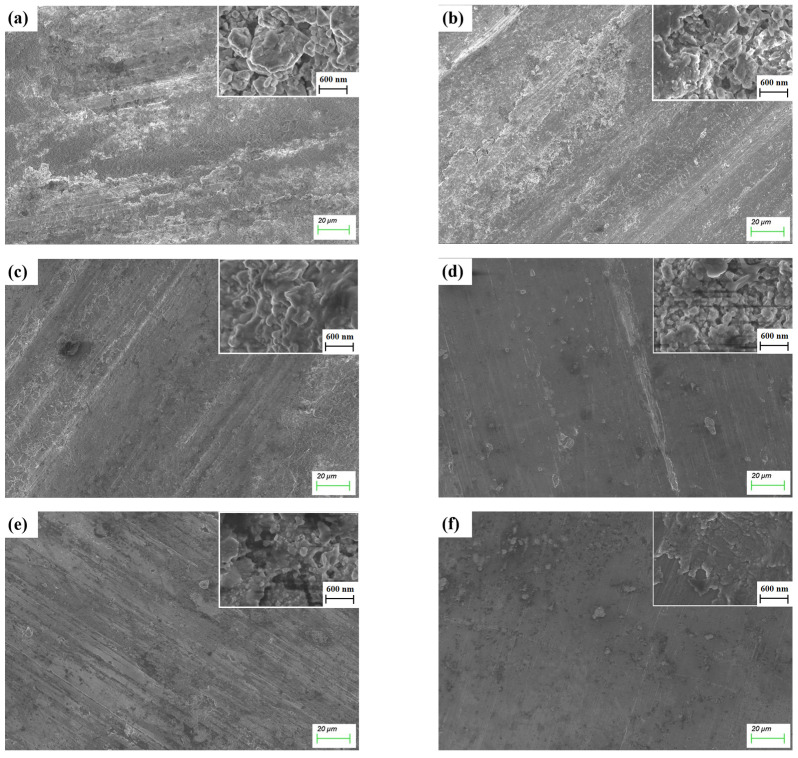
SEM images of Ti_3_SiC_2_-TiSi_x_ composites sliding against Al ball; (**a**) 2 GPa and dry sliding; (**b**) 4 GPa and dry sliding; (**c**) 4.5 GPa and dry sliding; (**d**) 2 GPa and wet sliding; (**e**) 4 GPa and wet sliding; (**f**) 4.5 GPa and wet sliding.

Figure 8 displays SEM images of wear tracks on virgin Ti_3_SiC_2_-TiSi_x_ composites sliding against an Al ball, as examined under an optical microscope. The variation in the scar area correlates with the changes in COF, as supported by optical microscope images. Under dry-sliding situations, the scar diameter of the Al ball increased from 1.966 mm to 2.536 mm before decreasing to 1.606 mm. Optical microscope images of Ti_3_SiC_2_-TiSi_x_ composites with Al balls are shown in Figure S2. The primary wear mechanisms of Ti_3_SiC_2_-TiSi_x_ composites against their Al counterparts involve pear groove wear and adhesive wear during dry sliding. The irregularly shaped Al chips accumulate in the pear grooves and adhere to one another. During wet sliding, pear groove wear predominates, while adhesive wear is significantly reduced. The abrasive chips are encapsulated by the lubricating fluid, leading to agglomeration within the medium.

**Figure 9:**
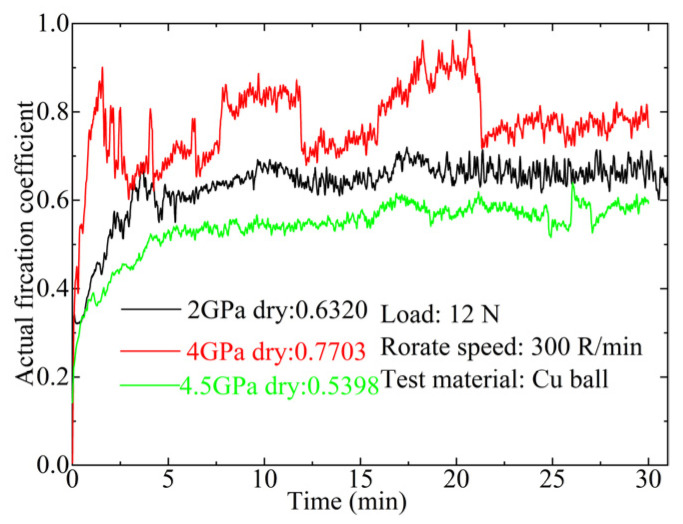
Typical measuring curves of the friction coefficients of Ti_3_SiC_2_-TiSi_x_ composites against the Cu ball pair at a load of 12 N and sliding speed of 0.05 m/s.

Figure 9 presents the typical measurement curves of the dynamic COF of Ti_3_SiC_2_-TiSi_x_ composites against Cu ball pairs under a 12 N load and a sliding speed of 0.05 m/s over a 30 min test duration. Following a 5 min running-in period, the COF of Ti_3_SiC_2_-TiSi_x_ composites sintered at 2 GPa and 4.5 GPa against the Cu ball pairs ranged from 0.5 to 0.7. In contrast, the Ti_3_SiC_2_-TiSi_x_ composites sintered at 4 GPa displayed a higher COF of 0.77, along with random fluctuation behavior. The COFs of Ti_3_SiC_2_-TiSi_x_ composites against Cu balls are lower than those of Ti_3_SiC_2_-ZnO composites tested against Inconel 78 alloy [15].

In the context of dry-sliding friction, researchers have identified two primary behaviors; transition behavior, where the COF shifts from an initial value to a steady state during the early phase of continuous friction, and random fluctuation behavior, characterized by turbulent variations in the entire friction process [43]. Different counterfaces exhibit varying frictional behavior under the same conditions, as compared to the Ti_3_SiC_2_-TiSi_x_/Al ball tribocouple. This suggests that the counterparts play a crucial role in the tribological performance of Ti_3_SiC_2_-TiSi_x_ composites.

**Figure 10:**
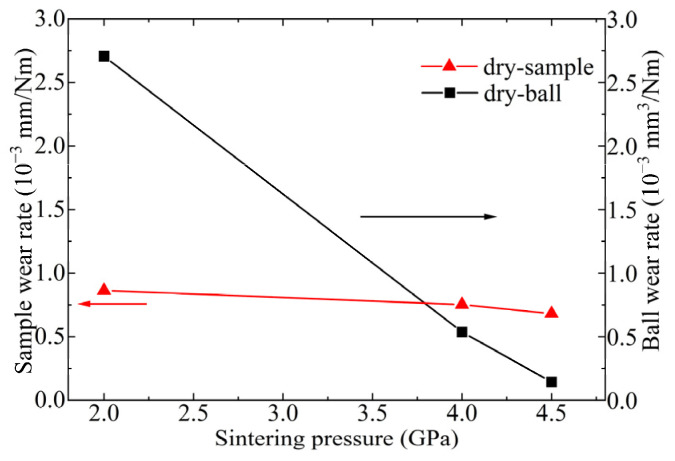
Variations in the wear rate of Ti_3_SiC_2_-TiSi_x_ composites against a Cu ball pair at a sliding speed of 0.05 m/s with a load of 12 N.

Figure 10 illustrates the wear rates of Ti_3_SiC_2_-TiSi_x_ composites against Cu ball pairs at a load of 12 N and a sliding speed of 0.05 m/s. The wear rate of Ti_3_SiC_2_-TiSi_x_ composites decreases with increasing sintering pressure. The wear rates of the Cu balls exhibited a similar trend.

**Figure 11:**
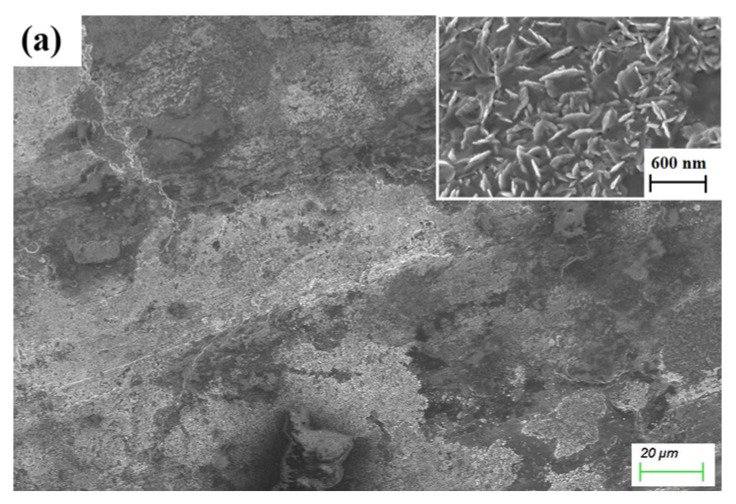

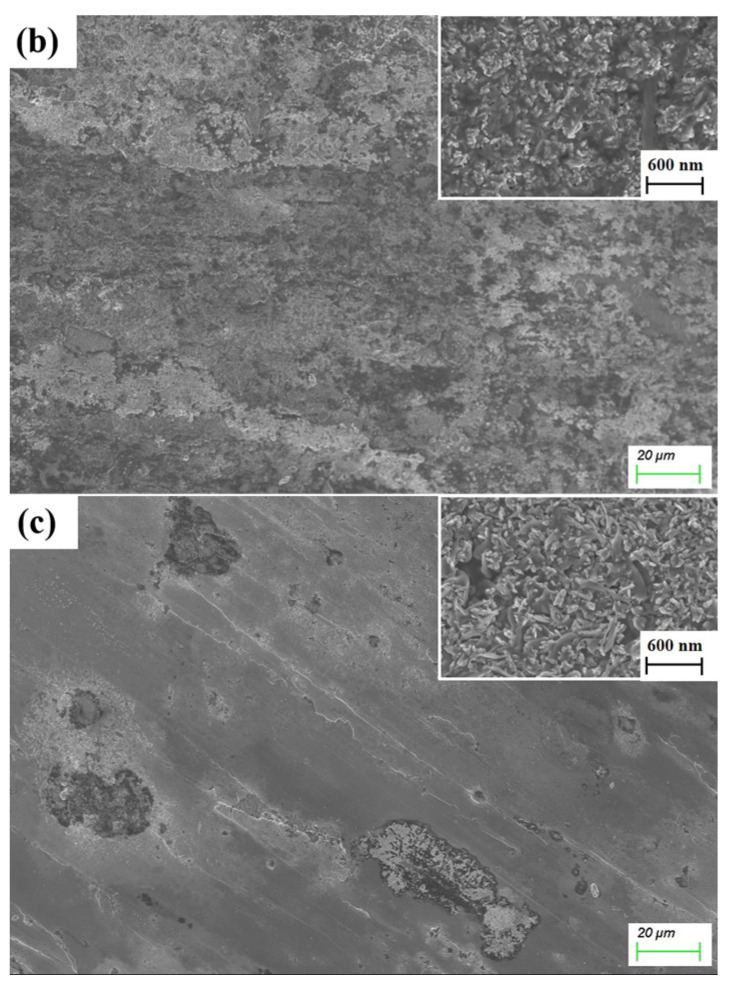
SEM images of Ti_3_SiC_2_-TiSi_x_ composites sliding against a Cu ball; (**a**) 2 GPa and dry sliding; (**b**) 4 GPa and dry sliding; (**c**) 4.5 GPa and dry sliding.

Figure 11 presents SEM images of wear tracks from virgin Ti_3_SiC_2_-TiSi_x_ composites sliding against Cu balls. As the sintering pressure rises, the scar length diminishes. The wear mechanism of the Ti_3_SiC_2_-TiSi_x_ composite in contact with its Cu counterpart includes pear groove wear and adhesive wear, with adhesive wear being more pronounced compared to the Al counterpart. The abrasive chips from the Cu balls are flakes that adhere to the contact interface. Optical microscope images of Ti_3_SiC_2_-TiSi_x_ composites paired with Cu balls are shown in Figure S3.

## 4. Discussion

Ti_3_SiC_2_ can be synthesized from Ti, Si/SiC, and graphite at 4 GPa and 1100 °C for varying soaking durations, as indicated by previous research [44]. At 2.0 GPa, the synthesis and formation mechanisms of Ti_3_SiC_2_ were investigated using the reactant species Ti/Si/C, Ti/SiC/TiC, Ti/SiC/C, Ti/SiC/C, and Ti/TiC/Si [20]. Ti_3_SiC_2_ forms at 1050 °C and 4.5 GPa, similar to the constituent combinations of Ti/Si/C/cBN, Ti/SiC/TiC/cBN, Ti/SiC/C/cBN, and Ti/TiC/Si/cBN [5]. If the initial elements are weighted according to the stoichiometric ratio, the presence of the impurity TiC becomes inevitable, as indicated by these findings [5,20,44]. A deficiency of Si promotes the formation of TiC, while an excess of Si favors the formation of TiSi_2_.

**Figure 12:**
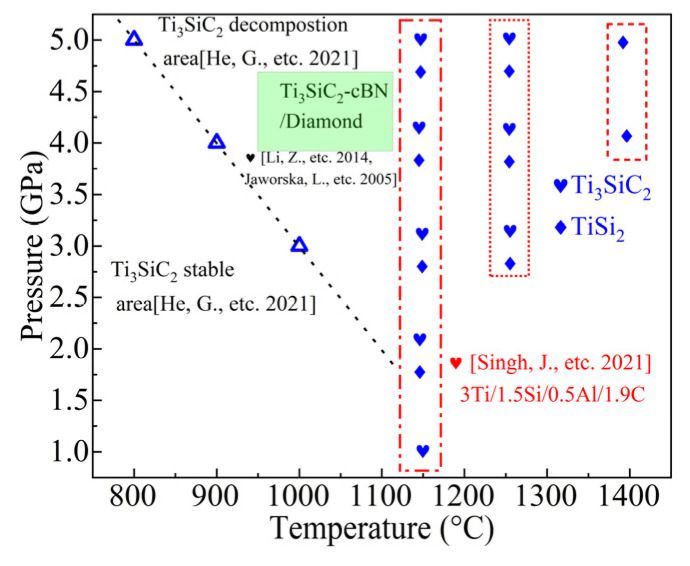
Comparison of stable areas of Ti_3_SiC_2_ [5,10,21] and Ti_3_SiC_2_-based composites.

In Figure 12, when Ti_3_SiC_2_ is employed as the raw material, the dotted line on the left divides the stable zone at high pressure. Ti_3_SiC_2_ decomposes into TiC as temperature and pressure rise. Ti_3_SiC_2_ composites can be synthesized at high pressures of 4.5 GPa or 5.5 GPa by combining titanium powder, silicon powder, carbon powder, or aluminum powder with a hard phase (such as diamond or cubic boron nitride). TiSi_2_ is used as the secondary phase enhancement in this study. TiSi_2_ serves as an intermediate in the synthesis of Ti_3_SiC_2_, helping to inhibit its decomposition. Additionally, Ti_3_SiC_2_-TiSi_x_ can be synthesized over a broader range of temperatures (1150–1250 °C) and pressures (3–5 GPa) compared to silicon enrichment. The presence of the second phase can enhance the stability of Ti_3_SiC_2_ by expanding its stable region at high pressure. The recommended parameters for the high-pressure synthesis of Ti_3_SiC_2_-TiSi_x_ are 4–5 GPa and 1400 °C. Considering the application purposes of previous studies on the superhard material binder, the excessive addition of too much Si to the Ti_3_SiC_2_ binder or Al to Ti_3_AlC_2_ may result in the formation of secondary phase TiSi_x_ alloys or TiAl_x_ alloys. It is meaningful to synthesize TiSi_x_-Ti_3_SiC_2_ as a superhard material bonding agent by varying the material ratios.

## 5. Conclusions

Ti_3_SiC_2_-TiSi_x_ composites were synthesized by a high-pressure and high-temperature method. The phase evolution of cermet Ti_3_SiC_2_ powder was studied under high pressure (1–5 GPa) and high temperature (1150–1400 °C). At 1 GPa and 1150 °C, high-content Ti_3_SiC_2_ is synthesized. Above 2 GPa, it transforms to TiSi_2_. In the middle-temperature zone (1250 °C), TiSi_x_ content increases with sintering pressure. The friction and wear properties of high-pressure synthetic Ti_3_SiC_2_ with aluminum and copper were investigated under normal temperature and dry/wet conditions. Under wet friction, the friction coefficient of Ti_3_SiC_2_-TiSi_x_ and Al balls is about 0.2. Under dry friction, it is comparable to that of Cu balls. Future research will focus on the high-temperature tribological properties and mechanisms of Ti_3_SiC_2_-TiSi_x_ composites with different ceramic counterparts for high-temperature composite ceramic applications.

## Data Availability

The data that support the findings of this study are available on reasonable request from the corresponding authors, Yuqi Chen and Liang Li.

## Supplementary Materials

The following supporting information can be downloaded at: https://www.mdpi.com/article/10.3390/ma17194866/s1, Figure S1: Variation in the error between the actual load value and the set value over time; Figure S2: Optical microscope images of Ti_3_SiC_2_-TiSi_x_ composites sliding against Al balls; Figure S3: Optical microscope images of Ti_3_SiC_2_-TiSi_x_ composites sliding against Cu balls.
