# Supplementary material for: Sintering and Tribological Properties of Ti3SiC2-TiSix Composite Sintered by High-Pressure High-Temperature Technology
Source: Materials (Basel). 2024 Oct 3;17(19):4866. doi: 10.3390/ma17194866 (PMC11477728; doi:10.3390/ma17194866)
Supplement: Supplementary file 1 [file materials-17-04866-s001.zip › materials-3202526-supplementary.pdf]

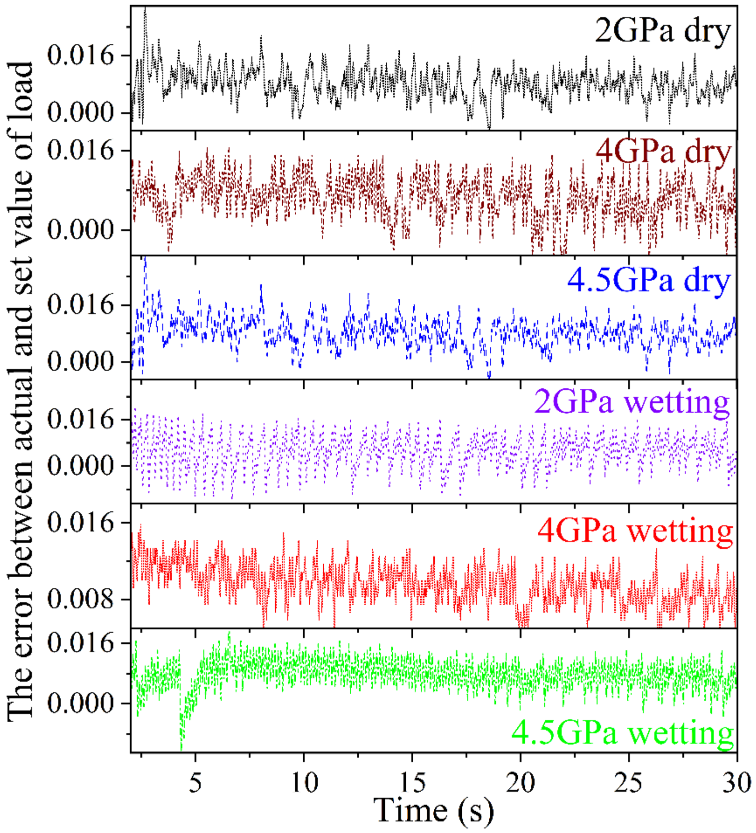

Figure S1. The variation of the error between the actual value and the set value of the load with time.

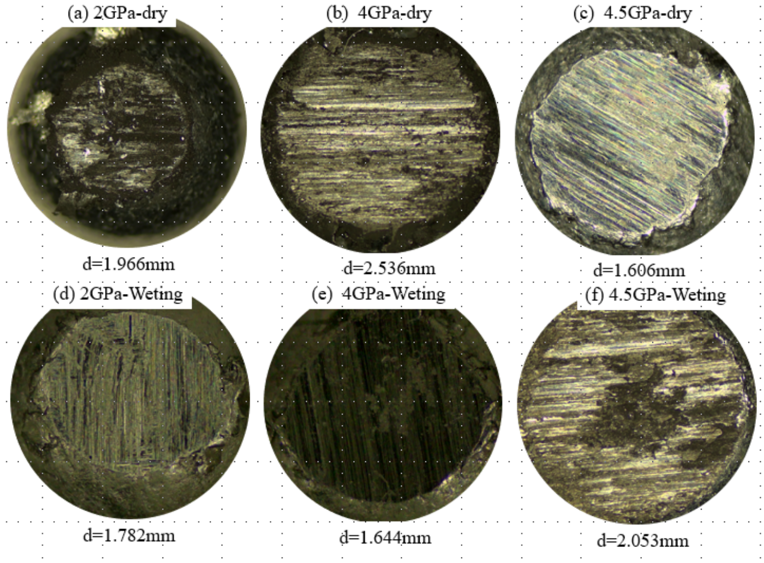

Figure S2. Optical microscope images of Ti_3SiC_2-$TiSi_x$ composites sliding against Al ball.

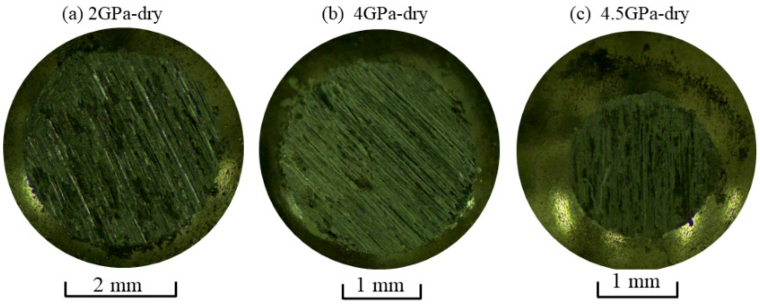

Figure S3. Optical microscope images of Ti_3SiC_2-$TiSi_x$ composites sliding against Cu ball.
